# Evaluation of continuous blood purification in patients with urosepsis caused by ureteral calculi and heart failure after catheterization

**DOI:** 10.12669/pjms.40.5.8386

**Published:** 2024

**Authors:** Xing Fan, Zhe Li, Yan Gao, Hai-song Zhang, Zhao-yu Bi

**Affiliations:** 1 Xing Fan, Department of Nephrology, Affiliated Hospital of Hebei University, Baoding 071000, Hebei, China; 2 Zhe Li, Department of Nephrology, Affiliated Hospital of Hebei University, Baoding 071000, Hebei, China; 3 Yan Gao, Department of Nephrology, Affiliated Hospital of Hebei University, Baoding 071000, Hebei, China; 4 Hai-song Zhang, Department of Nephrology, Affiliated Hospital of Hebei University, Baoding 071000, Hebei, China; 5 Zhao-yu Bi, Department of Internal Medicine, Affiliated Hospital of Hebei University, Baoding 071000, Hebei, China

**Keywords:** Continuous blood purification, Urosepsis, Heart failure, TNF-a, PCT, CRP

## Abstract

**Objective:**

To detect the continuous blood purification (CBP)’s application value in patients with urosepsis caused by ureteral calculi and heart failure after catheterization.

**Methods:**

This is a clinical comparative study. Sixty patients with ureteral calculi complicated with heart failure and urosepsis were admitted at Affiliated Hospital of Hebei University from January 2021 to March 2023 randomly split into control and experimental group(n=30). Based on conventional treatment after indwelling the DJ tube, the experimental group was treated with CBP therapy. The control group dealt with conventional anti-inflammatory, oxygen inhalation and other treatments only. Compared and analyzed in terms of alterations in blood inflammatory factors, cardiac function, BNP prior to and after therapy, blood pressure, blood WBC recovery time, and so on.

**Results:**

TNF-a, CRP, and PCT levels in the control and experimental groups were substantially more prominent than the average reference value prior to treatment. They decreased considerably at distinct time points after therapy, with substantial distinctions (p< 0.05). A more meaningful decrease was noticed in the experimental group in comparison with the control group (p< 0.05). BNP and cardiac function were improved in both groups prior to and after therapy, and the amelioration of indexes in the experimental group was more substantial than that in the control group after therapy, with statistically considerable distinctions. The improvement time in experimental group was earlier than in the control group, with statistically substantial differences.

**Conclusion:**

Patients with urosepsis complicated with heart failure after indwelling DJ tube have their inflammatory factors improved significantly, with more thorough excretion by using conventional treatment combined with CBP therapy.

## INTRODUCTION

Sepsis is an inflammatory syndrome caused by the body’s systemic inflammatory response and the release of a great many pro-inflammatory mediators and anti-inflammatory factors.^1^ Sepsis caused by urinary tract infections, one of the most common causes of sepsis in surgery, is generally called urosepsis, and it is usually a bacterial infection. Symptoms such as recurrent fever, chills, and anemia are common in patients with urosepsis, with multiple organs or tissues involved in the patient, and a high mortality rate.^2^

Some patients with urosepsis complicated with heart failure were still in critical condition after the obstruction was relieved by indwelling DJ tube and needed to be referred to ICU for further treatment. Extracorporeal blood purification technique has developed into an important treatment method in recent years, with an extraordinarily important role in the treatment of acute diseases such as sepsis.^3^ This technique has been applied in the further treatment of patients with urosepsis and heart failure after indwelling DJ tube, to detect the continuous blood purification (CBP)’s application value in patients with urosepsis caused by ureteral calculi and heart failure after catheterization.

## METHODS

This is a clinical comparative study. A total of 60 patients were included at Affiliated Hospital of Hebei University from January 2021 to March 2023 and split into two groups randomly, with 30 examples each. Seventeen females and 13 males were assigned to the experimental group, aged 32-68 years, with an average of 50.53±3.24 years old. Fourteen females and 16 males were assigned to the control group, aged 43-66 years old, with an average of 52.67±8.52 years old. No statistical distinction can be detected in the comparison of the general data of the two groups of patients, which are comparable (Table-I).

**Table-I:** Comparative analysis of general data between the two groups (*χ̅*±*S*) n=30.

	Experimental group	Control group	t/χ^2^	P
Male (%)	13(43%)	16(53%)	0.60	0.43
Age (years)	50.53±3.24	52.67±8.52	1.29	0.20
Cardiac function grade-III (%)	19(63%)	21(70%)	0.30	0.58

p>0.05.

### Ethical Approval:

The study was approved by the Institutional Ethics Committee of Affiliated Hospital of Hebei University on December 21, 2022(No.: HDFYLL-KY-2022-019), and written informed consent was obtained from all participants.

### Diagnostic criteria for ureteral calculi complicated with sepsis^4^

Body temperature > 38°C or < 36°C; (2) Heart rate > 90 beats/min; (3) Broken-winded, with respiratory frequency > 20 beats/minutes or over breathing, with PaCO2 < 32 mmHg; (4) White blood cell count > 12.0×109/L or < 4.0×109/L or immature white blood cell > 10%. Patients with two of the above four manifestations and accompanied by poor tissue perfusion or hypotension are diagnosed with ureteral calculi combined with sepsis. Hypoperfusion is manifested by symptoms such as oliguria, lactic acidosis, and acute changes in consciousness.

### Diagnostic standards for heart failure

As stated by the New York Heart Association (NYHA) Classification System^5^, cardiac function is divided into four grades, and heart failure is divided into three grades: Grade-I: mild dispersion of physical activity. No symptoms at rest, and daily activities may cause fatigue; Grade-II: mild limitation of physical activity, excessive fatigue, dyspnea or palpitation will not be caused by daily activities. Grade-III: obviously limited physical activity. No symptoms at rest, and mild daily activities will cause the above symptoms, also known as grade-II or moderate heart failure. Grade-IV: patients are unable to engage in any physical activity, and have congestive heart failure or angina pectoris symptoms at rest, which aggravates after any physical activity, also known as degree III or severe heart failure.

### Inclusion criteria:

- Patients with ureteral calculus complicated with sepsis and heart failure
- The course of heart failure < 48 hours, with cardiac function classification of Grade-III-IV (moderate and severe heart failure).
- Patients with successful ureteral stent drainage after surgical operation.

### Exclusion criteria:

- Patients with previous serious underlying diseases such as hypertension and diabetes.
- Patients with cardiac causes such as cardiomyopathy, severe valvular disease, coronary heart disease, myocarditis, etc.
- Patients with abnormal immune function or chronic inflammatory diseases.
- Patients with oral immunosuppressants and hormones.
- Patients with mental diseases or unable to complete their studies due to other reasons.
- Patients with failed ureteral stent placement and underwent other surgery.

### Stent placement method

Patients were locally anesthetized, sterilized and draped routinely in the lithotomy position. The ureteroscope was inserted into the bladder through the urethra. The zebra guide wire was inserted into the ureter after discovering the ureteral orifice of the affected side. The stent tube was guided into the ureter by the guide wire, with the position of the stent adjusted and the catheter indwelled. Abdominal X-ray film was performed immediately postoperatively to find out the position of the stent tube.

### Medical treatment

Conventional treatment was adopted in the control group after catheterization, including sensitive antibiotic control of infection, oxygen inhalation, nutrition therapy, cardiotonic, diuretic therapy, etc.; While the conventional treatment combined with CBP therapy was adopted in the experimental group: The German Fresenius multiFiltrate blood purification device was used to indwell a single-needle double-lumen catheter via internal jugular vein or femoral vein puncture. The CBP system was utilized, with a blood flow rate of 180 ML·min-1, a replacement rate of 2000 ML/h, a daily duration of about four to five h, and continuous treatment for seven days.

### Evaluation of serum inflammatory factors

Fasting blood was taken in the morning prior to treatment (before stent placement) and one, three and seven days after treatment (denoted as T1, T2, T3 and T4 respectively) to detect inflammatory factors TNF-A, CRP and PCT, and observe the changes of inflammatory factor levels between the two groups.

### Cardiac function evaluation

Prior to treatment (before stent placement) and seven days after treatment, cardiac index (CI) and left ventricular ejection fraction (LVEF) were measured by echocardiography^6^, and fasting venous blood was drawn in the morning to determine BNP quantification. A comparative analysis was conducted on the changes of cardiac function and BNP prior to and after treatment in the two groups.

### Sepsis treatment evaluation

Respiratory rate, heart rate, body temperature and other indexes of the two groups of patients at 6:00 am every morning and under basic condition were recorded respectively prior to treatment (before stent placement) and after treatment, and venous blood was drawn to detect white blood cell count. A comparative analysis was conducted on the time difference between the two groups in which each index returned to normal after treatment.

### Statistical methods

SPSS 25.0 software was used to analyze all the data statistically. The measurement data was presented as (*χ̅*±*S*). Paired t-test and two independent sample t-tests analyzed the data between the control and experimental groups, and the sample rates were compared using the χ^2^test. Data at different time points before and after each group’s therapy were analyzed by repeated measures data ANOVA. The confidence interval was 95%. P< 0.05 suggests the distinction is of statistical importance.

## RESULTS

It can be seen from the changes in serum inflammatory factors in the experimental group and the control group prior to and after treatment (Table-II) that the levels of TNF-a, CRP, and PCT in the two groups before treatment were significantly higher than the average reference values, with no significant difference between the two groups(p>0.05). After treatment, the levels of each group were significantly reduced at different time points, with a significant difference (p< 0.05). The reduction in the experimental group was more significant than that in the control group (p< 0.05), and the CRP between the two groups was significantly reduced after seven days of treatment, with no considerable difference (p= 0.15).

**Table-II:** Comparative analysis of inflammatory factors in the two groups prior to and after treatment (*χ̅*±*S*) n=30.

Group		T1*	T2Δ	T3Δ	T4	F	P
TNF-ɑ (ng/L)	Experimental groupΔ	45.52±12.81	23.33±11.24	6.37±1.24	4.32±2.12	16.13	0.00
	Control groupΔ	45.73±12.53	31.24±11.15	12.35±6.53	8.12±3.76	10.01	0.00
	*t*	0.64	2.74	5.69	4.82		
	*p*	0.94	0.01	0.00	0.00		
CRP (mg/L)	Experimental groupΔ	43.27±7.89	21.74±5.33	6.37±1.15	4.01±0.47	13.10	0.01
	Control groupΔ	43.56±8.06	32.15±7.43	15.82±3.41	4.32±1.07	10.59	0.02
	*t*	0.14	6.24	14.38	1.45		
	*p*	0.89	0.00	0.00	0.15		
PCT (ng/ml)	Experimental groupΔ	0.67±0.22	0.42±0.26	0.21±0.07	0.18±0.12	9.95	0.01
	Control groupΔ	0.64±0.27	0.57±0.21	0.33±0.10	0.27±0.14	10.04	0.00
	*t*	0.47	2.46	5.38	2.67		
	*p*	0.64	0.02	0.00	0.01		

* p> 0.05, Δp< 0.05.

BNP and the cardiac function of the two groups were improved prior to and after treatment, with a significant difference(p=0.00). The experimental group exhibited more substantial amelioration in each index after therapy than the control group, and the distinction was statistically important (CI=0.01, LVEF=0.02, BNP=0.00) (Table-III).

**Table-III:** Comparative analysis of cardiac function and BNP in the two groups prior to and after treatment (*χ̅*±*S*) n=30.

Observation index	Cardiac index (CI) L/min.m^2^3-3.5				Left ventricular ejection fraction (LVEF) %55-80				BNP (pg/ml)			

Group	Prior to treatment*	After treatmentΔ	t	p	Prior to treatment*	After treatmentΔ	t	p	Prior to treatment*	After treatmentΔ	t	p
Experimental groupΔ	1.93±0.47	3.32±0.34	13.12	0.00	0.35±0.07	0.62±0.14	9.45	0.00	923.57±231.23	99.76±13.45	19.51	0.00
Control groupΔ	1.87±0.57	3.02±0.21	10.37	0.00	0.33±0.05	0.53±0.15	6.93	0.00	916.28±245.14	146.52±43.37	16.95	0.00
*t*	0.44	4.11			1.27	2.40			0.11	5.73		
*p*	0.65	0.01			0.21	0.02			0.91	0.00		

* p> 0.05, Δp< 0.05.

The two groups of patients had significant improvements in vital signs, respiration, heart rate, body temperature and other indexes after treatment, among which the improvement time of the experimental group was earlier than that of the control group, and the difference was of statistically significance (respiration p=0.01; heart rate and body temperature p=0.00). The WBC recovery in the experimental group was faster than that in the control group, with a statistically significant difference (p=0.00) (Table-IV).

**Table-IV:** Comparative analysis of clinical symptoms of sepsis in the two groups prior to and after treatment (*χ̅*±*S*) n=30.

Index	Respiration (d)*	Heart rate (d)*	Body temperature (d)*	WBC (d)*
Experimental group	2.53±0.52	2.16±0.94	2.08±0.61	2.04±0.42
Control group	3.27±0.80	3.79±0.69	3.47±0.61	3.02±0.36
*t*	4.25	7.66	8.83	9.70
*p*	0.01	0.00	0.00	0.00

* p<0.05.

## DISCUSSION

It was confirmed in this research that prior to therapy, the degrees of TNF-a, CRP and PCT in the control group and the experimental group were considerably more eminent than the normal values, with no meaningful distinction between the two groups(p>0.05). Whereas after treatment, a substantial decrease is visible in these degrees in each group compared to those prior to therapy. These degrees ameliorated more considerably in the experimental group (p< 0.05), implying that CBP possesses a sounder impact on eradicating inflammatory factors.

CBP has obvious advantages in protecting myocardium and improving cardiac function. It was confirmed in a study involving 434 patients that the long-term poor prognosis will not be caused by new left ventricular dysfunction in patients with sepsis and septic shock.^7^ It is therefore of great help to the prognosis of patients to remove blood endotoxins and avoid further damage to myocardium. In this study, the two groups of patients were confirmed to have improved CI, LVEF, and BNP prior to and after treatment, in which more substantial improvement is visible in the experimental group in comparison with the control group, with statistically considerable distinctions (CI= 0.01, LVEF= 0.02, BNP= 0.00). BNP can reach 1000 ng/L or more in patients with sepsis complicated with heart failure.

This study’s consequences provide patients’ diagnoses with an essential clinical reference with sepsis complicated with heart failure, and patients’ heart participation can be monitored. A decrease in BNP helps judge the prognosis, and a favorable prognosis[a rapid decrease in BNP often indicates.^8^ More than 19 million people suffer from sepsis each year, which is defined as life-threatening acute organ dysfunction secondary to infection, such as infection and heart failure that affect each other and promote its exacerbation.^9^ Cardiac hypofunction is often caused by sepsis because sepsis affects the calcium ion transport function of myocardial cells, resulting in reduced contractility, enlarged heart, and reduced stroke volume and left ventricular ejection index.^10^

Furthermore, problems such as vasodilation, increased permeability, insufficient blood volume and ventricular dysfunction may be caused by various inflammatory factors.^11^ The incidence of sepsis complicated with heart failure is about 23.5%, which is an important cause of death.^12^ Therefore, timely correction of cardiac function, improvement of microcirculation and rapid reduction of inflammatory mediators in patients with sepsis are of great importance for the treatment and prognosis of sepsis.

The filter has in vitro adsorption properties on blood-derived cytokines and other humoral media of sepsis, and can quickly reduce its accumulation in the body.^13^ In patients receiving uninterrupted blood purification, blood degrees of inflammatory cytokines including tumor necrosis factor-α, IL-6 and IL-8 and procalcitonin were discovered to be substantially lower than those in the conventional treatment group.^14^ Other researches have confirmed that CBP exerts its parts in considerably converting the degrees of procalcitonin(PCT) and other inflammatory markers in patients with septic shock prior to and after treatment.^15^

The greater impact will be caused by the elimination of inflammatory factors and toxins on the improvement of patient symptoms, and a certain guiding value will be provided by the level of white blood cell changes on the severity of the disease and the treatment effect.^16^ It was confirmed in this study that after treatment, the improvement time of vital signs, respiration, heart rate, body temperature and other indexes of the experimental group was earlier than that of the control group (respiration p=0.01; heart rate and body temperature p= 0.00). The WBC recovery in the experimental group was faster than that in the control group, with a statistically significant difference (p= 0.00). According to a meta-analysis of CBP with 11 studies included and 679 patients recruited, mortality could be significantly reduced via CBP in patients with sepsis or ARDS.^17^ Meanwhile, attention should be paid to improving microcirculation^18^ and ensuring hemodynamic stability^19^ in patients who have sepsis complicated with heart failure.

### Limitations

Nevertheless, deficiencies still exist in this study: **1.** No more detailed individualized treatment plan is designed according to the recovery of patients. **2.** The sample size is small and needs to be further increased. **3.** All patients were discharged from the hospital after their condition was under control, and re-visited the clinic one month later for lithotripsy and then the treatment ended, which was lack of long-term follow-up. Further research summaries are ongoing to analyze the long-term benefits of the operation to patients.

## CONCLUSION

Patients with urosepsis complicated with heart failure have their inflammatory factors improved significantly, with more thorough excretion by using conventional treatment combined with CBP therapy. Such a favorable regimen is more conducive to the recovery of cardiac function and the improvement of clinical symptoms. It is believed that for critically ill patients, CBP should be carried out as soon as possible after the DJ tube is inserted. Early CBP helps to significantly improve the prognosis of sepsis.^20^

### Authors’ Contributions:

**XF** and **ZL:** Designed this study and prepared this manuscript, and are responsible and accountable for the accuracy or integrity of the work.

**YG** and **HZ:** Collected and analyzed clinical data.

**ZB:** Significantly revised this manuscript.
